# Application of exosomes derived from human umbilical cord stem cells in the treatment of ovalbumin-induced allergic asthma in mice

**DOI:** 10.1186/s42826-026-00280-y

**Published:** 2026-05-18

**Authors:** Yong Ding, Yanli Zhang, Qi Cao, Yong Yin, Zhenglai Ma, Wenxia Pan, Xinjie Du, Chunling Ma

**Affiliations:** 1 Key Laboratory of Birth Defect Prevention and Control, Women’s and Children’s Health Care Hospital of Linyi, Linyi, Shandong 276000 China; 2 Department of Medical Laboratory, Shandong Pharmaceutical and Food Vocational College, Weihai, Shandong 264210 China; 3 Laboratory Center of Molecular Biology, Shandong Medical College, Linyi, Shandong 276000 China

**Keywords:** Human umbilical cord mesenchymal stem cells, Exosomes, Allergic asthma, Mouse models, Budesonide

## Abstract

**Background:**

This study investigated the therapeutic potential of exosomes derived from human umbilical cord mesenchymal stem cells (hUCMSCs-exos) in an Ovalbumin (OVA) -induced mouse model of allergic asthma, comparing their efficacy to budesonide, a standard asthma treatment. hUCMSCs-exos were characterized by their typical morphology, size, and marker expression (CD9, CD63, and HSP70) using transmission electron microscopy (TEM), Nanosight 300, and Western blot analysis.

**Results:**

Treatment with hUCMSCs-exos significantly decreased lung inflammation by detecting plasma IL-4 and IgE levels, as well as white blood cells and eosinophils counts in Bronchoalveolar lavage fluid (BALF), the M1 and M2 type of macrophages, Regulatory T Cells (Tregs) in lung tissues, paralleling the effects of budesonide therapy. Notably, hUCMSCs -exos achieved a greater reduction in the percentage of eosinophils in BALF and a significant increase M2 macrophages and Tregs in lung tissues.

**Conclusions:**

These findings suggest hUCMSCs-exos may offer comparable or superior therapeutic benefits to budesonide, warranting further investigation into their molecular mechanisms to support clinical application.

## Background

Asthma, a chronic inflammatory disease of the airways, affects individuals of all ages and is characterized by recurrent symptoms such as wheezing, breathlessness, chest tightness, and cough [1]. Globally, the World Health Organization estimates approximately 300 million asthma patients, with prevalence rates varying significantly across countries. Wales (18%), New Zealand (15%), and Ireland (13.5%) report the highest prevalence [2–4]. In China, asthma prevalence differs by age group, with 4.2% in adults aged 20 years and older and 3.02% in children [5].

Allergic asthma, a subtype of asthma, is a chronic inflammatory disorder involving various cellular components, including eosinophils, mast cells, T lymphocytes, neutrophils, and airway epithelial cells [6, 7]. This condition is often associated with a Th2-dominant immune response, characterized by elevated levels of cytokines such as IL-4, IL-5, and IL-13, along with increased serum immunoglobulin E (IgE) and eosinophilia [8, 9]. Although treatments like budesonide, a glucocorticoid with anti-inflammatory and anti-allergic properties, are widely used to manage allergic asthma, they are associated with significant side effects when used long-term. These include hypertension, diabetes, osteoporosis, acute gastroenteritis, and oral candida infections from inhaled corticosteroids [10–13].While mesenchymal stem cells (MSCs) have shown promise in preclinical studies for treating lung injuries and pulmonary inflammation, their application faces practical challenges. These include difficulties in preservation, transportation, and potential risks such as immune rejection and tumorigenesis [14].

Exosomes, small extracellular vesicles (30–200 nm in diameter), offer a promising alternative. Derived from MSCs, exosomes serve as carriers of bioactive molecules, mediating intercellular communication and exerting potent immunomodulatory effects. These vesicles reduce inflammation, promote tissue repair, and regulate immune responses, making them highly relevant for conditions like asthma, characterized by immune dysregulation and chronic inflammation [15–19]. Compared to live MSCs, exosomes present several advantages: (i) Enhanced Safety: Exosomes eliminate the risk of tumorigenesis associated with stem cell therapy.(ii)Practicality: They are more stable, easier to store, and do not require complex preservation or transportation systems. (iii)Targeted Action: Their ability to carry specific bioactive molecules enables more precise therapeutic effects. (iv)Low immunogenicity: The possibility of causing immune rejection is relatively low. (v)low in ethical controversy. It does not raise ethical controversy like traditional cell therapy.

As a non-cellular therapeutic modality, exosomes also avoid the systemic side effects of glucocorticoids therapy, such as those associated with glucocorticoids, while effectively mitigating allergic asthma-related inflammation [20–22]. These advantages highlight the necessity of developing exosome-based therapies to improve asthma treatment outcomes.This study applied hUCMSCs-exos to treat allergic asthma in mouse models. The research aimed to establish a foundation for their clinical application by evaluating their safety, efficacy, and optimal dosage. The findings could pave the way for exosome-based therapies as an alternative to conventional treatments for allergic asthma.

## Materials and methods

### Materials

#### Human umbilical cord tissue specimens

This study was approved by the Ethics Committee of the Women’s and Children’s Health Care Hospital of Linyi (Approval No. KYL-YXLL-2021025). We affirm that the research adhered to the principles outlined in the 1964 Declaration of Helsinki and it subsequent amendments. Human umbilical cord tissue specimens obtained with approval from the Ethics Committee of the Women’s and Children’s Health Care Hospital of Linyi and informed consent from donors, were used as the source materials.

#### Animal

The animal experiments in this study were in accordance with the guidelines and study protocols of the Institutional Animal Care and Use Committee of Women^,^s and Children^,^s Health Care Hospital of Linyi (approval no. [2021]002). All animal housing and experiments adhered to the institutional guidelines for the care and use of laboratory animals. Male BALB/C mice aged 6–8 weeks were obtained from Beijing Vital River Laboratory Animal Technology Co., Ltd. (Beijing, China).

### Methods

#### Extraction and culture of hUCMSCs

Human umbilical cord tissue specimens were provided by the Women’s and Children’s Health Care Hospital of Linyi. This study was approved by the hospital’s Ethics Committee, with informed consent obtained from donors. Fresh umbilical cords were rinsed twice with phosphate-buffered saline (PBS) containing penicillin and streptomycin (Gibco, Carlsbad, CA). The two arteries and one vein were removed, and the remaining tissue was cut into approximately 1 mm³ fragments, which were then attached to culture plates.

The tissue fragments were enzymatically digested with 1 mg/mL collagenase I and 10 mg/mL hyaluronidase at 37 °C for 1 h. After digestion, the cell suspension was filtered through a 40 μm cell sieve and cultured in stem cell culture medium (Cyagen, Guangzhou, China) at 37 °C with 5% CO₂. The medium was replaced every 3 days, and fibroblast-like colonies appeared after approximately 10 days. These colonies were passaged into new plates for expansion. hUCMSCs from passages 3–5 were used in subsequent experiments. The experimental protocol was approved by the hospital’s Ethics Committee.

#### Identification method for hUCMSCs

After the third passage, the hUCMSCs were harvested using trypsinization and washed twice with PBS. Multipotency of osteocyte, adipocyte, and chondrocyte differentiation of hUCMSCs cultured in the differentiation media. The cells were analyzed using cytochemical staining with Alizarin Red, Oil red O, and Alcian Blue, respectively. The cells were then stained with the following human-specific antibodies: anti-CD105, anti-CD73, anti-CD90 (all antibodies were obtained from BD Biosciences Pharmingen, San Jose, CA). Flow cytometric analysis was performed using a FACSVerse instrument (BD Biosciences), and at least 10,000 events were recorded per sample. The data were subsequently analyzed using FlowJo software (Tree Star, Ashland, OR).The experimental protocol was approved by the Women^,^s and Children^,^s Health Care Hospital of Linyi Ethics Committee.

#### Preparation and characterization of hUCMSC-exos

The method was adapted from [23]. Briefly, the hUCMSCs culture supernatant was centrifuged at 300×g for 10 min to remove suspended and dead cells. An additional centrifugation at 2000×g for 10 min was performed to eliminate cellular debris and other impurities. The supernatant was then filtered using 0.22 μm sterile filters. Exosomes were precipitated by ultracentrifugation at 100,000×g for 120 min at 4 °C. The resulting pellets were washed, resuspended in PBS, and aliquoted for storage at -80 °C to preserve their integrity for subsequent applications.

1. Exosomes were visualized using transmission electron microscopy with a negative staining method. Approximately 10 μL of the exosome suspension was applied to a copper mesh grid and left for 3 minutes at 4°C. The grid was then washed, blotted dry with filter paper, and negatively stained with 1% phosphotungstic acid for 2 minutes. The samples were examined using a transmission electron microscope (JEM-1200EX, JEOL) operating at 120 kV, and images were captured using a digital camera (Soft Imaging Solution, Olympus).
2. For particle size and concentration analysis, the NanoSight NS 300 system (NanoSight Technology, Malvern, UK) was used. The sample was diluted to ensure single nanoparticle tracking. Video capture and data analysis were performed to determine the particle size distribution and concentration (particles/mL).
3. The protein concentration in exosomes was determined using the BCA protein concentration determination kit (Thermo, Shanghai, China). For the method, see the manufacturer's manual.

Proteins from hUMSC-exos were extracted and quantified using a BCA Protein Assay Kit (Beyotime). Equal amounts of lysed exosomes (50 µg) were separated by electrophoresis on a 10% sodium dodecyl sulfate-polyacrylamide gel (SDS-PAGE) and transferred onto polyvinylidene difluoride (PVDF) membranes (Bio-Rad, Hercules, CA) pre-soaked in 100% methanol. Non-specific binding was blocked with Tris-buffered saline containing 0.05% Tween-20 (TBST) and 5% non-fat milk powder, followed by incubation with primary antibodies. The CD63 monoclonal antibody (cat#ab134045), CD9 monoclonal antibody (cat#ab263019), and HSP70 monoclonal antibody (cat#ab181606) were all purchased from Abcam (Cambridge, MA). To detect positive markers (CD9,CD63 and HSP70) and the purity of huMSC-exos, Calnexin monoclonal antibody (Cat#66903-1-1 g, Proteintech, Wuhan) was stained as a negative control.The blots were then washed with TBST and incubated with an HRP-linked anti-rabbit secondary antibody (cat#ab6728; Abcam) for 1 h at room temperature. Finally, immunoblot signals were visualized using ECL chemiluminescence kit (Millipore, Billerica, MA) and imaged using a ChemiScope 3400 Mini (CLINX Science Instruments, Shanghai, China).

#### Construction of mouse models of allergic asthma and intervention therapy

All mice randomly allocated into five groups (*n* = 5). Four groups received OVA to induce allergic asthma models, while the fifth group served as the normal control. The three of the four OVA-induced asthma model groups were treated with hUCMSCs-exos, budesonide, or PBS, respectively.

The specific methods for asthma model construction and treatment were as follows:

Mice were sensitized via intraperitoneal injection of 0.2 mL of a mixture containing OVA and AL(OH)₃ on days 1 and 14. The sensitized mice were subsequently exposed to 5% OVA aerosol for 30 min daily from day 21 to day 27 in a plexiglass chamber to induce asthma. The normal control group (CON) underwent PBS aerosolization using the same method.

The budesonide treatment group received aerosolized budesonide (10 µg/mL) using mouse aerosol inhalers for 10 minutes, twice daily, over seven consecutive days. The hUCMSCs-exos treatment group received 1 mL of hUCMSCs-exos aerosolized therapy at protein concentration: 0.70 mg/mL and at particle counts 1.45 × 10^10^ particles/mL for 10 minutes, twice daily, for seven consecutive days [24]. hUCMSCs-exos were derived from the culture supernatant of hUMSCs obtained from the same healthy donor.

Following modeling or treatment, venous blood was collected using the periocular venous plexus sampling method. After euthanasia via CO₂ inhalation, mouse lung tissues and BALF were collected. IgE and IL-4 levels in venous blood were determined using ELISA. Lung tissue sections were stained using the HE method, and total white blood cells and eosinophils in BALF were quantified.

#### Collection of BALF and measurement of total white blood cells and eosinophils in BALF and measurement of IL-4 and IgE in plasma

Within 24 h of the final stimulation on day 28, the trachea was dissected under sterile conditions. After ligating the left main bronchus, the right main bronchus was cut, a tracheal catheter was inserted and fixed, and the lung was irrigated three times with sterile saline (1 mL each time).The collected BALF was centrifuged at 4 °C and 1200 rpm for 5 min. The supernatant was discarded, and the cell pellet was resuspended in PBS buffer. Total white blood cells in BALF were counted using semiconductor laser flow cytometry combined with nucleic acid fluorescence staining. Smears were prepared, and the percentage of eosinophils in BALF was determined using Wright’s staining. Blood samples were collected from the orbital vein of the mice. IgE and IL-4 concentrations in plasma were measured following the instructions provided in the ELISA kit (Elabscience, Wuhan, China).

#### Lung histopathology

After BALF collection, the left lung tissues were fixed in 10% neutral-buffered formalin (48 h) and embedded in paraffin fixation. Then, the paraffin-embedded Sect. (4 μm thick, 3 sections per animal, 5 mice for each group) were stained with HE to evaluate the lung inflammatory. Inflammation score in the lungs were performed in a blind-way [24], inflammation was graded as follows: grade 0 (no inflammatory cells were observed), grade 1 (inflammatory cells were occasionally observed), grade 2 (bronchi were surrounded by 1–3 layer of inflammatory cells), grade 3 (bronchi or vessels were surrounded by 4–5 layer of inflammatory cells), and grade 4 (most bronchi or vessels were surrounded by more than 5 layer of inflammatory cells). At least 8 bronchioles were counted in each slide and then the mean inflammation score was calculated for each mouse.

Lung tissue samples from exosomes and budesonide-treated and control mice were collected, and total RNA was extracted for transcriptomic analysis. RNA sequencing (RNA-seq) was performed using the Illumina platform followed by alignment to the mouse reference genome using STAR and gene expression quantification with feature Counts. Normalized expression data were analyzed using single-sample Gene Set Enrichment Analysis (ssGSEA) with the GSVA R package to estimate the relative abundance of immune cell populations. Immune cell gene sets were obtained from ImmuneSigDB sources. Enrichment scores for macrophages and Tregs were compared between groups using statistical tests, and the results were visualized with boxplots to evaluate differences in immune cell infiltration following treatment.

#### Statistical analysis

Statistical analysis was performed using SPSS Statistics Version 26.0. The normality of the data was assessed using the Shapiro-Wilk test, while homogeneity of variance was tested using Levene’s test. Measurement data that met the assumptions of normality and homogeneity of variance were analyzed using one-way ANOVA, with pairwise comparisons conducted using the Tukey-Kramer method. For data that did not meet these assumptions, group comparisons were performed using the Kruskal-Wallis H test.

To account for multiple comparisons and reduce the risk of Type I errors, post-hoc corrections were applied where appropriate. Specifically, for pairwise comparisons following ANOVA, the Tukey-Kramer post hoc tests was used. Data were considered statistically significant for P values less than 0.05. Statistical plots were generated using Prism 9.0 software.

## Results

### Characterization and multipotency assessment of hUCMSCs

**Fig. 1:**
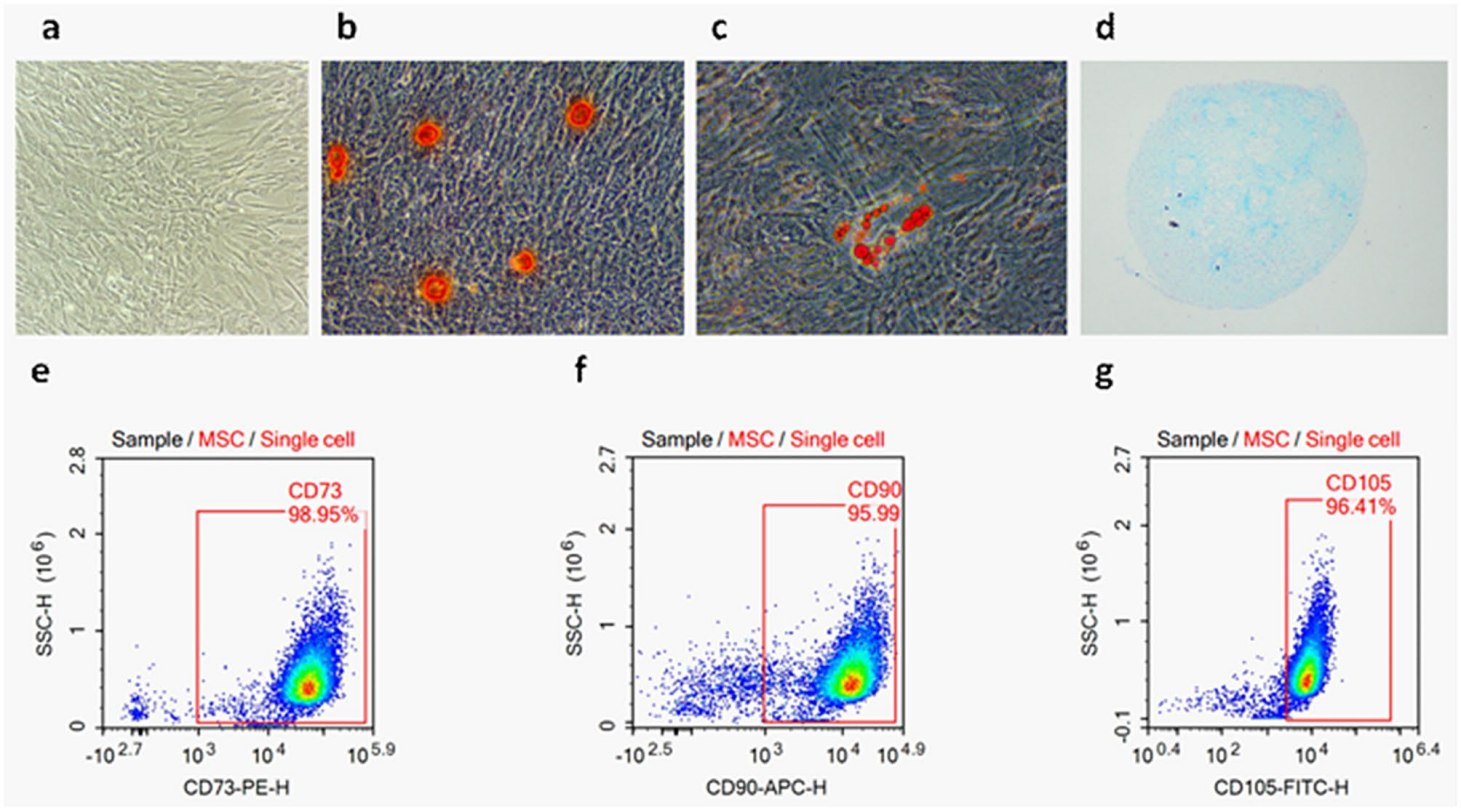
Characterization of hUCMSCs. **a**. The cell morphology of hUCMSCs (passage 3) was observed under a light microscope (magnification, ×100). **b**,** c**,**d.** Representative images of osteocyte (B, ×100), adipocyte (C, ×200), and chondrocyte (D, ×40) differentiation of hUCMSCs cultured in differentiation media. The cells were analyzed using cytochemical staining with Alizarin Red (**b**), Oil Red O (**c**), and Alcian Blue (**d**), respectively. **e**,** f**, **g**. Flow cytometric analysis showed the expression of MSC-related cell surface markers: CD90 (95.99%), CD105 (96.41%), and CD73 (98.95%)

The hUCMSCs were purified and confirmed on the basis of the criteria defined by International Society for Cellular Therapy [25]. These minimal criteria of MSCs include(i) the ability to self-renew, (ii) multipotency with osteogenic, chondrogenic, and adipogenic potentials, and (iii) expression of a characteristic set of surfacemarkers, such as cluster of differentiation CD73, CD90, and CD105 are positive. In current study, the self-renewal ability of the hUCMSCs, the differentiation potential of osteoblasts, lipoblasts and chondroblasts were all positive(Fig. 1a, b, c and d). In the meantime, flow cytometry was used to detect characteristic the hUCMSCs markers, such as CD73, CD90, and CD105. Their expression levels were as follows, CD73 was 98.95%, CD90 was 95.99%, CD105 was 96.41%, respectively (Fig. 1e, f and g).

### Characterization of exosomes isolated from hUCMSCs

**Fig. 2:**
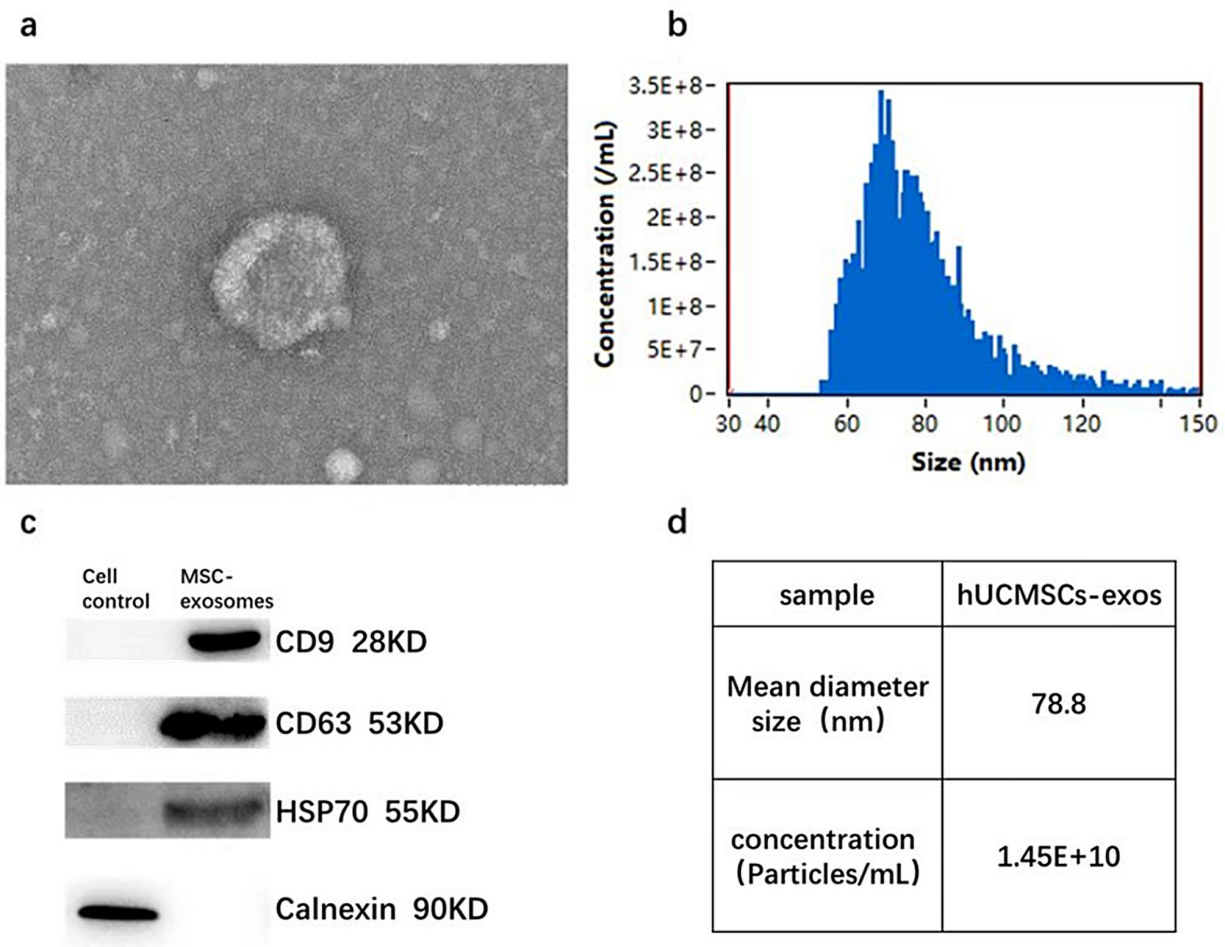
Identification of hUCMSC-exos. **a**. The morphology of hUCMSC-Exos was observed using transmission electron microscopy (scale = 100 nm). **b**. The diameter distribution of exosomes was assessed through nanoparticle tracking analysis using Nanosight 300. **c**. Exosome surface marker expressions were analyzed using Western blot. **d**. The Nanosight 300 quantified the concentration, mean diameter of particles of exosomes isolated from the hUCMSC supernatant

Exosomes isolated from hUCMSCs were round or oval in shape, with intact membranes and typical exosomal morphology, as observed under transmission electron microscopy (Fig. 2a). The hUCMSC-Exos were analyzed using the Nanosight 300, revealing a concentration of 1.45 × 10¹^0^ particles/mL (Fig. 2d). The particle size ranged from about more than 50 nm to 150 nm, the mean diameter size is 78.8 nm, consistent with the reported dimensions of exosomes (Fig. 2d). Western blot analysis of hUCMSC-Exos showed significant expressions of CD9, CD63, and HSP70 (Fig. 2c).For Western blot identification, the protein concentration of exosomes was determined by BCA method and the protein concentration is 0.70 mg/ml.

### hUCMSC-exos treatment reduces plasma IL-4 and IgE levels in asthma model mice

**Fig. 3:**
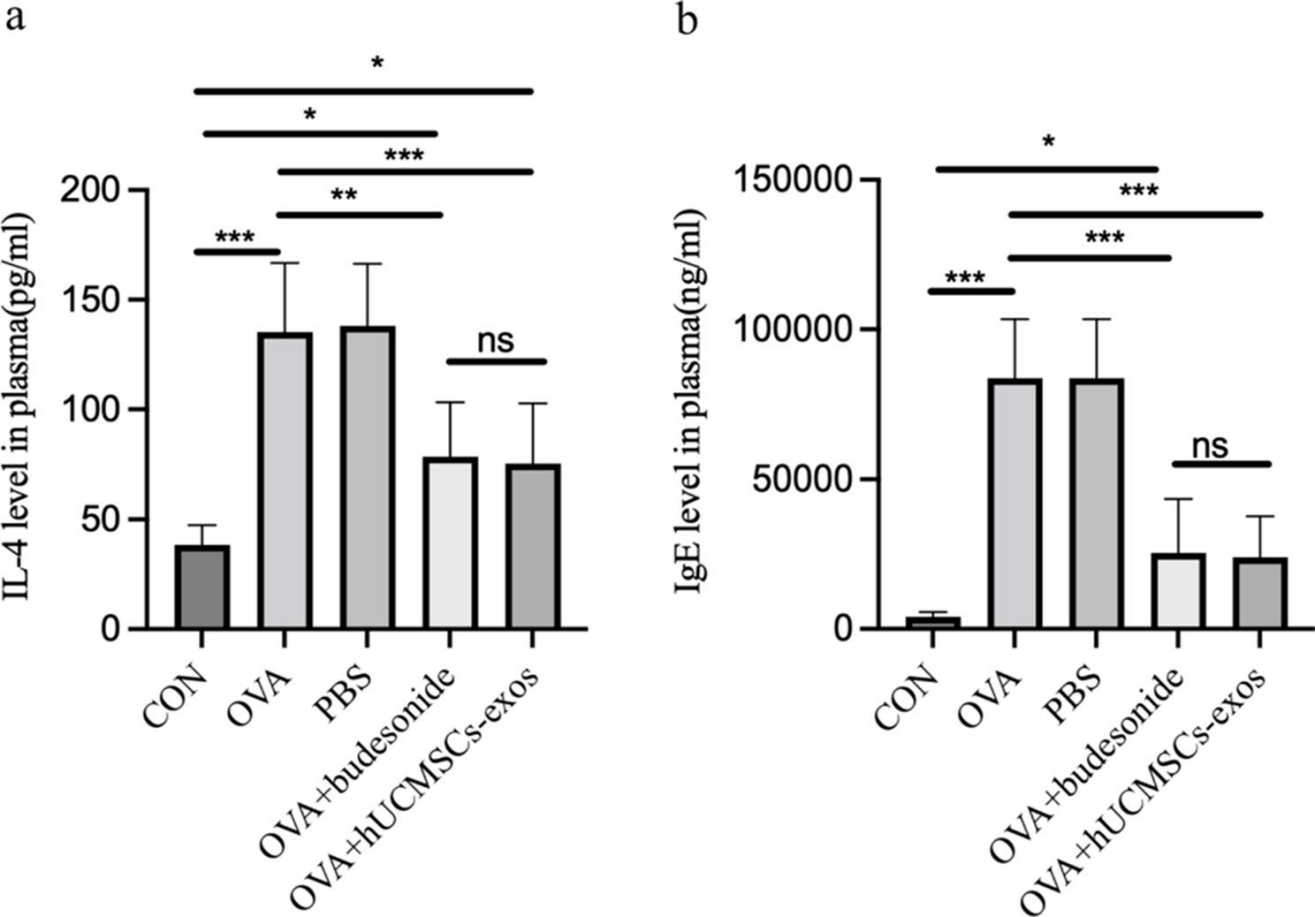
hUCMSC-exos treatment reduced the IL-4 and IgE levels in mouse plasma. After hUCMSC-exos inhalation treatment, plasma IL-4 and IgE levels of each group were detected by ELISA. **a**. Statistical analysis of the IL-4 levels in the plasma.ELISA analysis confirmed IL-4 expression increase in the model group (P = 0.0104, P = 0.0071), and the PBS-treated group (P = 0.00006), compared to the normal control group. Treatment with hUCMSC-exos (P = 0.0164, P = 0.0112) significantly reduced IL-4 levels, compared to the model group, or the PBS-treated group, and no differece to comparable to Budesonide (P = 0.9996). **b**. The IgE levels in the plasma (n = 5). IgE concentrations were significantly lower in the normal control group but markedly elevated in the asthma model (P = 0.000002) and PBS-treated groups (P = 0.000002). Treatment with hUCMSC-exos (P = 0.000087, P = 0.000087) or Budesonide (P = 0.000123, P = 0.000121) significantly reduced IgE levels compared to the model and PBS-treated groups. IgE reduction in the hUCMSC-exos group was comparable to that in the Budesonide group (P = 0.999870, ns, Fig. 3b). CON (normal control group); OVA (asthma model control group); PBS (PBS treatment group), OVA+budesonide (budesonide treatment group); OVA+hHUMSCs-exos (hHUMSCs-exos treatment group).*P < 0.05, **P < 0.01,***P < 0.001, ns: not significant

Allergic airway inflammation is characterized by elevated IL-4, a key pro-inflammatory cytokine produced by Th2 cells, and increased IgE, which mediates type I hypersensitivity [26]. As expected, IL-4 levels were significantly higher in the asthma model group compared to the normal control, which exhibited minimal IL-4 expression. ELISA analysis confirmed this increase in the model group (*P* < 0.05, *P* < 0.01, respectively), and the PBS-treated group (*P* < 0.001),compared to the normal control. Treatment with hUCMSC-exos (*P* < 0.05, *P* < 0.05, respectively) significantly reduced, compared to the model group or the PBS-treated group, and similarly to Budesonide treatment (P > 0.05,ns). These results suggest that hUCMSC-exos effectively alleviate airway inflammation by lowering IL-4 levels (Fig. 3a).

IgE concentrations were significantly lower in the normal control group but markedly elevated in the asthma model (*P* < 0.001) and PBS-treated groups (*P* < 0.001). Treatment with hUCMSC-exos (*P* < 0.001, *P* < 0.001, respectively) or Budesonide (*P* < 0.001, *P* < 0.001, respectively) significantly reduced IgE levels compared to the model and PBS-treated groups. Notably, IgE reduction in the hUCMSC-exos group was comparable to that in the Budesonide group (*P* > 0.05, ns, Fig. 3b).These findings demonstrate that hUCMSC-exos effectively reduce IL-4 and IgE levels in OVA-induced asthmatic model mice, highlighting their potential as a therapeutic strategy for allergic airway inflammation.

### hUCMSC-exos treatment reduces inflammatory cell infiltration in BALF

Bronchial asthma is primarily categorized based on inflammatory cell infiltration, including eosinophilic, neutrophilic, mixed-type, and oligocellular asthma, the latter lacking typical inflammatory cell involvement. Eosinophilic asthma is the most prevalent, accounting for approximately 80% of cases. To assess airway inflammation, we measured the total number of white blood cells and eosinophils in BALF.

**Fig. 4:**
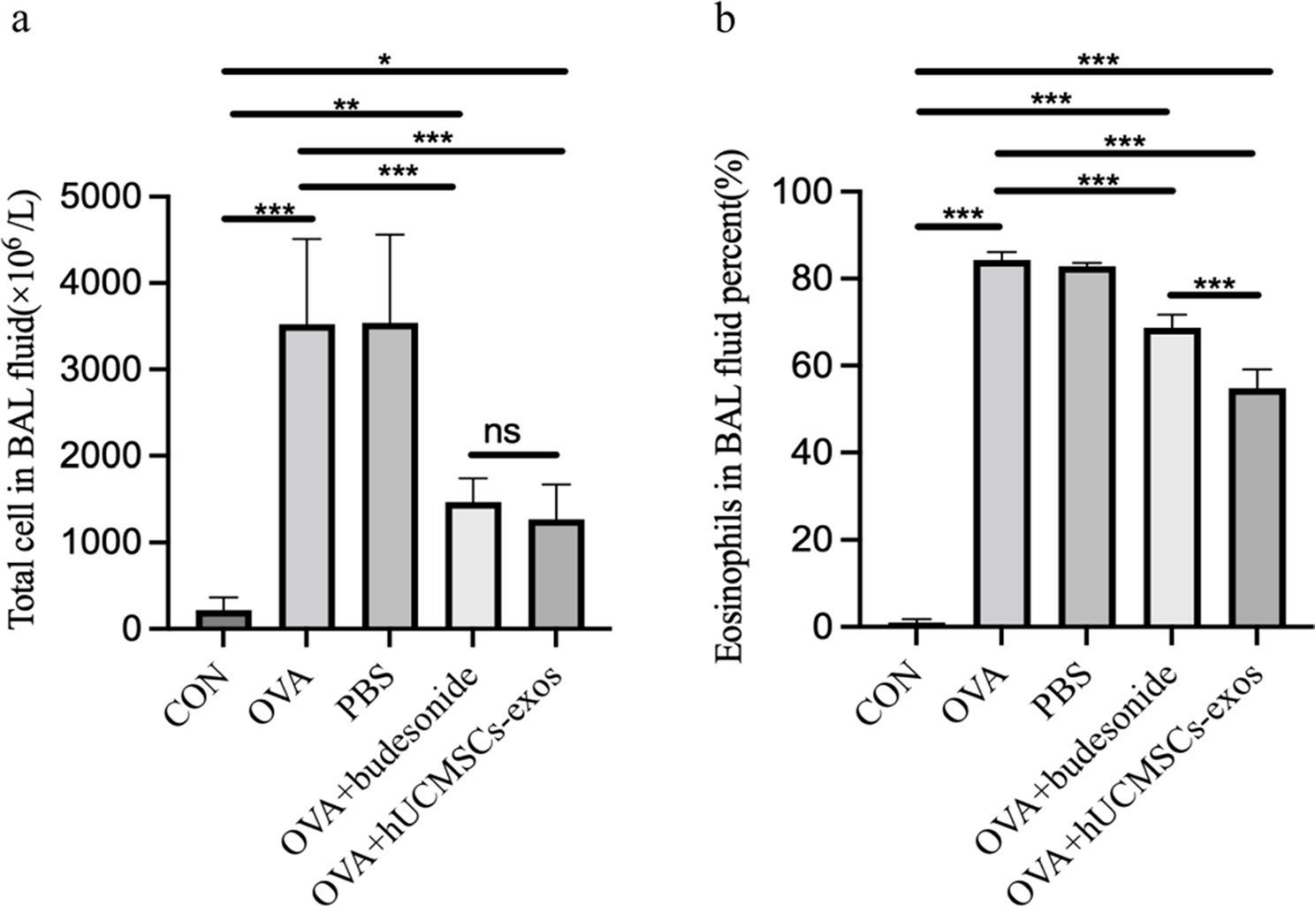
hUCMSC-Exos treatment reduced inflammatory cell infiltration in BALF. Statistical analysis of total inflammatory cell counts in BALF. (**b**) Statistical analysis of eosinophil counts in BALF (n = 5). Total inflammatory cell counts were measured using semiconductor laser flow cytometry with nucleic acid fluorescence staining, while eosinophil percentages in BALF were determined using Wright’s staining.The total inflammatory cell and eosinophil counts were significantly elevated in both the model group (OVA) (P < 0.000002, P = 0.000031) or the PBS-treated group (P = 0.000002, P = 0.000012) compared to the normal control. Treatment with hUMSC-exos(P = 0.000281, P = 0.000025) and Budesonide(P = 0.000311, P = 0.000001), significantly reduced these cell counts compared to the PBS-treated group and the OVA-induced model group(Fig. 4a and b). *P < 0.05, **P < 0.01, ***P < 0.001, ns: not significant

In OVA-induced asthmatic mice, the total inflammatory cell and eosinophil counts were significantly elevated in both the model group (OVA) (*P* < 0.001) or the PBS-treated group (*P* < 0.001) compared to the normal control. Treatment with hUMSC-exos(*P* < 0.001, *P* < 0.001, respectively)and Budesonide(*P* < 0.001, *P* < 0.001, respectively), significantly reduced these cell counts relative to the PBS-treated group and the OVA-induced model group (Fig. 4a and b).

Furthermore, while both treatments similarly decreased total inflammatory cell numbers, with no significant difference between the two groups (*P* > 0.05,ns), eosinophil reduction was significantly greater in the hUMSC-exos group compared to the Budesonide group (*P* < 0.001). These findings suggest that hUMSC-exos effectively mitigate airway inflammation in OVA-induced asthma and may offer therapeutic benefits comparable to or exceeding those of Budesonide.

### Histopathological evaluation of the therapeutic effect of hUCMSC-Exos

#### Assessment of inflammatory cell infiltration by HE Staining

To evaluate the therapeutic effect of hUMSC-exos, an asthmatic mouse model was established through intraperitoneal injection of a mixture of OVA and aluminum hydroxide (AL[OH]₃) for 14 consecutive days. Mice treated with hUMSC-exos received aerosolized hUMSC-exos at 1mL each time(the protein concentration is 0.70 mg/mL) twice daily for seven consecutive days from day 21 to day 27 [24]. Control groups included mice treated with Budesonide or PBS, as well as untreated mice.

**Fig. 5:**
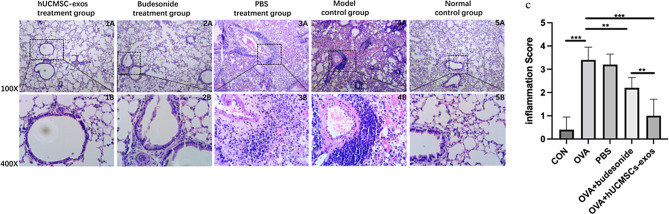
Effects of hUCMSC-Exos Treatment on inflammatory infiltration in mice with OVA-Induced allergic asthma. Section photographs of 1 A and 1B at different magnifications were the same representative results derived from the hUCMSCs-exos treatment group. 2 A and 2B from the budesonide treatment group. 3 A and 3B from PBS treatment group. 4 A and 4B from model control group. Figure 5A and B from normal control group. **C**. The inflammatory infiltration were quantified by HE scores performed in a blind-way(n = 5). In the hUMSC-exos-treated mice was notably lower compared to the model (p = 0.000062) and PBS groups (p = 0.000005), similar to those observed in the Budesonide treatment group (p = 0.002534, p = 0.003246). *P < 0.05, **P < 0.01, ***P < 0.001

Histological analysis using HE staining revealed that OVA-challenged mice (model control group)and mice treated with PBS (PBS treatment group) exhibited significant peribronchial and perivascular inflammatory cell infiltration compared to the normal control group. Treatment with hUMSC-exos or Budesonide significantly reduced this allergic inflammation compared to the asthma model control group and PBS-treated group. Furthermore, the inflammatory score in the hUMSC-exos-treated mice was notably lower compared to the model (*p* < 0.001) and PBS groups (*p* < 0.001), these results similar to those observed in the Budesonide treatment group (*p* < 0.01, *p* < 0.01, respectively). The hUCMSCs-exos treatment group exhibited significant improvement, different to the lung tissue status of the Budesonide treatment group (*p* < 0.01), with the most pronounced effect being a reduction in inflammatory cell infiltration.These findings suggest that hUMSC-exos effectively attenuate chronic airway inflammation(Fig. 5).

### Macrophage and Tregs infiltration in lung tissues

Lung tissue samples from exosome-treated, Budesonide-treated, and control mice were collected, and total RNA was extracted for transcriptomic analysis. Enrichment scores for macrophages and Tregs were compared between groups using statistical tests, and the results were visualized with boxplots to assess differences in immune cell infiltration following treatment.

Macrophages play a critical role in allergic inflammation, contributing to pathogen clearance, phagocytosis, and tissue homeostasis. They are classified into M1 macrophages, which promote inflammation, and M2 macrophages, which exhibit anti-inflammatory properties, each functioning under distinct pathological conditions.

**Fig. 6:**
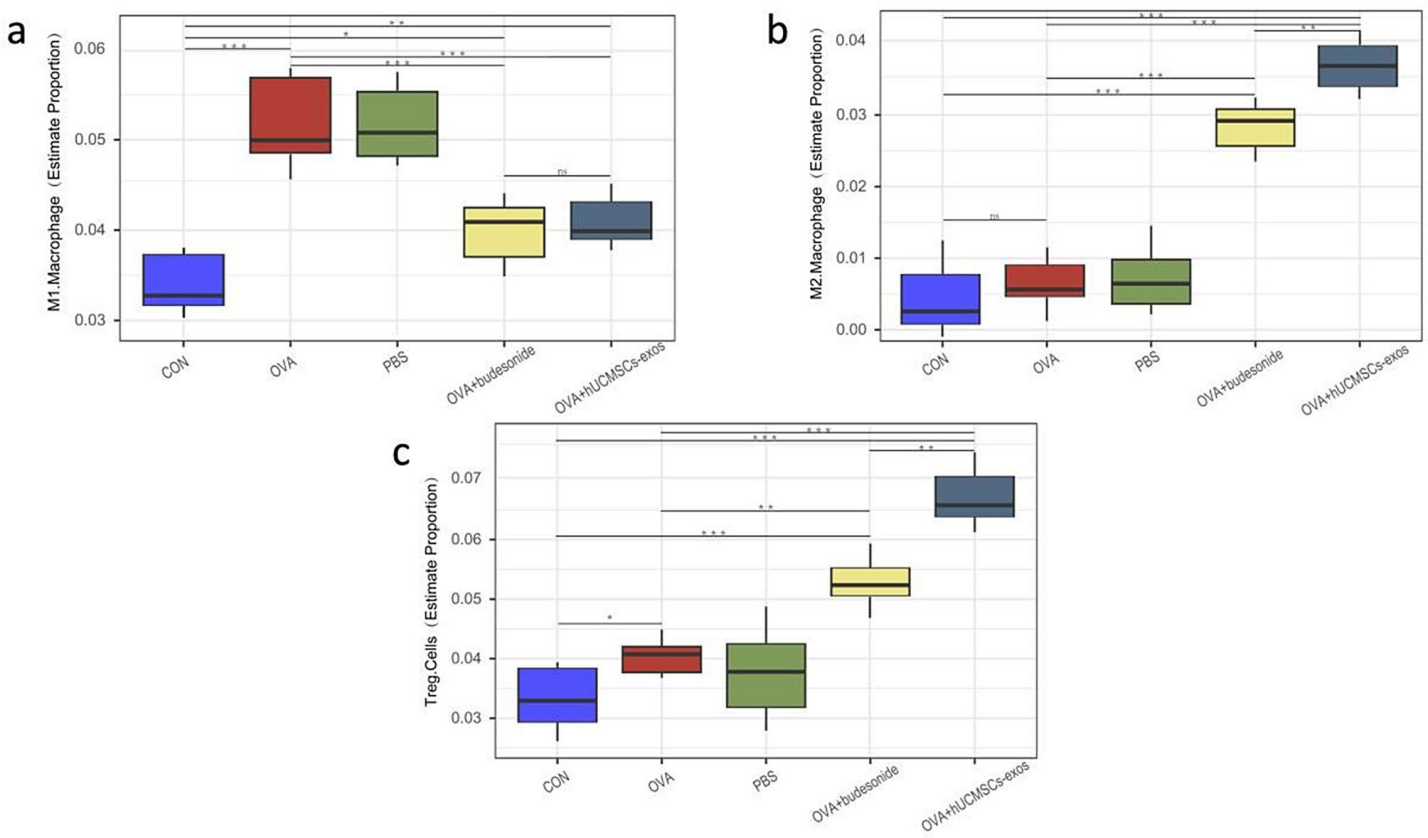
Transcriptomic analysis of M1 and M2 macrophage and Treg infiltration in lung tissues. After transcriptomic analysis, M1 macrophage proportion increased significantly in the model group (P = 0.0000023), or PBS-treated group (P= 0.0000056) compared to the normal control. Treatment with hUMSC-Exos (P =0.000013, P=0.000025) and Budesonide (P = 0.000062, P = 0.000034) markedly reduced compared to the PBS-treated or OVA-induced model group, respectively (Fig.6a). For M2 macrophages, the proportions remained comparable between the model (P = 0.14260), or PBS-treated (P = 0.18560), and normal control groups. both hUMSC-Exos (P = 0.000034, P = 0.000016) and Budesonide (P = 0.00014, P = 0.00016) significantly increased compared to the PBS-treated and OVA-induced model groups (Fig.6b). For Tregs cells,compared to the normal control group, the proportion in the model group (p=0.034051) and the PBS treatment group (p=0.043202) were increased. hUMSC-exos (p=0.000014, p=0.000032) and Budesonide (p=0.004432, p=0.004681) administration dramatically increased, compared with the PBS treatment group or the OVA induced model group, respectively(Fig.6c). *P < 0.05, **P < 0.01, ***P < 0.001

In this study, M1 macrophage levels were significantly elevated in the model group (*P* < 0.001) and PBS-treated group (*P* < 0.001) compared to the normal control. Treatment with hUMSC-Exos (*P* < 0.001, *P* < 0.001, respectively) and Budesonide (*P* < 0.001, *P* < 0.001, respectively) markedly reduced M1 macrophage proportions compared to the PBS-treated and OVA-induced model groups. However, no significant difference was observed between the hUMSC-Exos and Budesonide treatment groups (*P* > 0.05, Fig. 6a).

For M2 macrophages, their proportion remained comparable between the model (*P* > 0.05), PBS-treated (*P* > 0.05), and normal control groups. However, both hUMSC-Exos (*P* < 0.001, *P* < 0.001, respectively) and Budesonide (*P* < 0.001, *P* < 0.001, respectively) significantly increased M2 macrophage levels compared to the PBS-treated and OVA-induced model groups. Notably, hUMSC-Exos treatment resulted in a greater M2 macrophage increase than Budesonide (*P* < 0.01,Fig. 6b).

Tregs can secrete anti-inflammatory cytokines, mainly IL-10 and TGF-β, which inhibit the functions of antigen-presenting cells and effector T cells, and play a role in regulating immune response and anti-inflammatory pathology. In this study, compared to the normal control group, the proportion of Tregs cells in the model group (*p* < 0.05) and the PBS treatment group (*p* < 0.05) were increased. hUMSC-exos (*p* < 0.001, *p* < 0.001, respectively) or Budesonide (*p* < 0.01, *p* < 0.01, respectively) administration dramatically increased the Tregs proportion in lung, compared with the PBS treatment group or the OVA induced model group, respectively. In addition, compared to the OVA challenged mice treated by Budesonide, hUMSC-exos treatment increased the Tregs cell proportion(*p* < 0.01), and the difference were found between the two treatment groups (Fig. 6c).

### The comparison of the differences between the hUCMSCs-exos and budesonide treatment for the asthma model mice

**Fig. 7:**
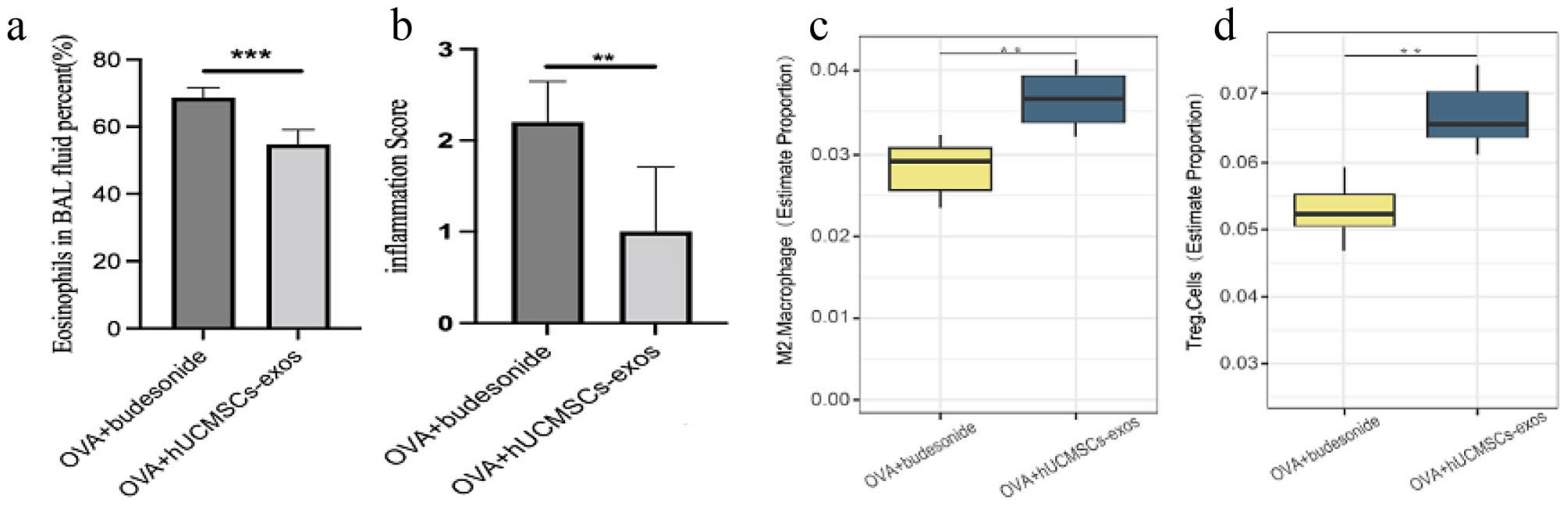
The differences between the hUCMSCs-exos and budesonide treatment for the asthma model mice. Compared to budesonide, hUCMSCs-exos treatment for asthma in this study appears more effective by detecting BALF, HE staining and Transcriptomics analysis. a In the hUMSC-exos treatment group a significant decrease in eosinophil numbers compared to the Budesonide group (P = 0.000003, Fig. 7a). b HE staining indicated a significant reduction in inflammatory cell infiltration compared to the Budesonide group (P = 0.005003, Fig. 7**b**). **c**, **d** After transcriptomic analysis, M2 macrophages and Treg cells in the hUCMSC-exos group were significantly higher than in the Budesonide group (P = 0.001530, Fig. 7c; P = 0.001430, Fig. 7d). *P < 0.05, **P < 0.01, ***P < 0.001

Statistical analysis revealed a significant decrease in eosinophil numbers in the hUMSC-exos treatment group compared to the Budesonide group (*P* < 0.001, Fig. 7a). HE staining indicated notable improvements in lung tissue morphology following hUCMSC-exos treatment, with a significant reduction in inflammatory cell infiltration compared to the Budesonide group (*P* < 0.01, Fig. 7b).

Further analysis of immune cell populations showed that the proportion of immunosuppressive M2 macrophages in the hUCMSC-exos group was significantly higher than in the Budesonide group (*P* < 0.01, Fig. 7c). Similarly, the level of immunosuppressive Treg cells was also elevated in the hUCMSC-exos group compared to the Budesonide group (*P* < 0.01, Fig. 7d).

Overall, this study demonstrated that hUCMSC-exos treatment reduced eosinophil infiltration in BALF and inflammatory cell accumulation in lung tissue, while increasing the presence of M2 macrophages and Treg cells, compared to Budesonide. These findings suggest that hUCMSC-exos provide superior therapeutic effects in alleviating chronic airway inflammation induced by OVA in mouse lungs, surpassing the efficacy of Budesonide treatment.

## Discussion

Allergic asthma is a significant global health issue, with an increasing incidence rate of approximately 5% annually. It is characterized by an immune-inflammatory response predominantly mediated by type 2 T helper lymphocytes (Th2), resulting from complex interactions between the innate and adaptive immune systems. OVA-induced allergic airway inflammation models are widely used to study Th2 cytokine-mediated processes, such as eosinophilic recruitment, IgE production, and goblet cell proliferation [27]. Among these, interleukin-4 (IL-4), secreted by Th2 cells, B cells, and mast cells, plays a pivotal role in IgE synthesis, a key mediator of type I hypersensitivity. Elevated levels of IL-4 and IgE correlate with increased allergen sensitization and severity of asthma [28–33].

In this study, hUCMSCs-exos demonstrated significant therapeutic effects in allergic asthma mouse models by reducing inflammatory cell accumulation, eosinophil infiltration, and cytokine release. These findings align with reports that MSC-derived exosomes possess immunomodulatory capabilities, influencing both innate and adaptive immune responses. For instance, hUCMSCs-exos have been shown to upregulate IL-10 and TGF-β1 secretion from peripheral blood mononuclear cells (PBMCs), thereby enhancing Treg proliferation and immunosuppressive activity, which are critical in mitigating asthma-associated inflammation [34, 35].

A distinctive feature of exosomes is their ability to mediate intercellular communication through bioactive cargo, such as proteins, lipids, and microRNAs (miRNAs). Emerging evidence highlights the role of specific miRNAs in MSC-derived exosomes in regulating asthma immunopathogenesis. For example, miR-21 and miR-146a are implicated in Th2 cytokine modulation, while miR-126 has been shown to influence eosinophilic recruitment [36, 37]. M2 macrophages can play an anti-inflammatory role in asthma through exosomes. M2-derived macrophage-derived exosomes carrying miR-370 have been shown to alleviate asthma progression in OVA-induced asthmatic mice by down-regulating FGF1 expression and MAPK/STAT1 signaling pathways [38].In Addition, Scorpion and centipede alleviates evere asthma through M2 macrophage-derived exosomal miR-30b-5p has been reported [39].These mechanisms suggest that the therapeutic effects of hUCMSCs-exos may be partially attributed to their miRNA contents, which warrants further investigation.

Compared to other non-steroid therapies for asthma, such as leukotriene receptor antagonists and monoclonal antibodies (e.g., anti-IgE and anti-IL-5 therapies), exosome-based treatments offer unique advantages. Exosomes provide a cell-free therapeutic approach that avoids the risks associated with live cell therapies, such as tumorigenesis and immune rejection, while also being more stable and easier to store and transport. Additionally, exosomes can be engineered to enhance their specificity and efficacy, offering a versatile platform for targeted therapies [38–40].

The advantages of aerosolized inhalation for exosome delivery were also considered. The lung’s extensive alveolar surface area, thin cell membranes, and rich capillary network facilitate rapid drug absorption while minimizing systemic side effects. Compared to systemic delivery routes, inhalation allows for smaller dosages and localized action, which is particularly advantageous for respiratory diseases like asthma [41, 42].

While budesonide remains a cornerstone in asthma management due to its potent anti-inflammatory effects, its long-term use is associated with significant side effects, including hypertension, diabetes, osteoporosis, and oral candida infections [43, 44].In contrast, hUCMSCs-exos offer comparable or superior efficacy without these adverse effects, as demonstrated in this study.The hUCMSC-exos treatment reduced eosinophil infiltration in BALF and inflammatory cell accumulation in lung tissues, while increasing the presence of M2 macrophages and Tregs, compared to Budesonide. These findings suggest that hUCMSC-exos provide superior therapeutic effects in alleviating chronic airway inflammation induced by OVA in mouse lungs, surpassing the efficacy of Budesonide treatment.

Moreover, their ability to reduce eosinophilic inflammation and modulate immune responses—specifically macrophage polarization and Treg cell numbers—without the complications associated with glucocorticoid therapy highlights their potential as a safer alternative for long-term asthma management.

This study has several limitations that need further investigation. First, the molecular mechanisms behind the therapeutic effects of hUCMSC-exosomes in allergic asthma remain unclear, requiring more detailed studies. Second, the impact of aerosolized delivery on exosome integrity and function should be thoroughly assessed, as atomization may affect their bioactivity. Third, although efficacy was demonstrated, a detailed histological analysis of immune cell infiltration in lung tissues was not performed. Additionally, potential off-target effects of hUCMSC-exosomes, including unintended distribution to non-target tissues, should be studied. Lastly, the long-term safety of hUCMSC-exosomes, especially with repeated or high-dose administration, must be evaluated through longitudinal studies to assess potential risks like immunogenicity and chronic toxicity. Addressing these gaps will be essential for establishing the safety, efficacy, and clinical application of hUCMSC-exosomes in treating allergic asthma.

## Conclusions

While these findings highlight the potential of hUCMSCs-exos as a promising alternative to traditional asthma therapies, the study’s implications must be interpreted with caution. The lack of detailed mechanistic insights and clinical validation limits the generalizability and immediate applicability of these results. Further studies are needed to elucidate the molecular mechanisms underlying the observed effects, optimize delivery methods, and evaluate long-term safety and efficacy in clinical settings. By addressing these gaps, future research can better establish the role of hUCMSCs-exos in asthma treatment and facilitate their translation into clinical applications. This study provides an important step toward exploring exosome-based, cell-free therapies for allergic asthma.

## Data Availability

All data generated or analyzed during this study are included in this published article.

## Abbreviations

ANOVA: Analysis of Variance
AL[OH]₃: Aluminu mhydroxide
BALF: Bronchoalveolar lavage fluid
BCA: Bicinchoninic Acid
ECL: Electrochemiluminescence
ELISA: Enzyme-linked immunesorbent assay
FGF1: Fibroblast growth factor-1
GSVA: Gene Set Variation Analysis
hUCMSCs: Human umbilical cord MSCs
hUCMSC-exo: Exosomes derived from hUCMSCs
HSP70: Heat shock protein 70
HE: Hematoxylin and eosin
IgE: Immunoglobulin E
IL: Interleukin
MSCs: Mesenchymal stem cells
M1 or M2: Macrophages
miRNAs: microRNAs
MAPK: Mitogen-Activated Protein Kinase Signaling Pathway
OVA: Ovalbumin
PBS: Phosphate-buffered saline
PBMCs: Peripheral blood mononuclear cells
PVDF: Polyvinylidene Difluoride
STAT1: Signal Transducer and Activator of Transcription 1
ssGSEA: Single-sample Gene Set Enrichment Analysis
TBST: Tris-buffered saline containing 0.05%
Tregs: Regulatory T Cells
TGF-β1: Transforming growth factor-β1
Th2: Type 2 T helper lymphocytes
TEM: Transmission electron microscope
